# Correspondence

**Published:** 1868-08-01

**Authors:** E. R. Hutchins

**Affiliations:** Philadelphia


### *CORRESPONDENCE.*

PHILADELPHIA, *July* 14, 1868.

*To the Editor of the Chicago Medical Journal :*

CASE 1.—J. W. ; age 59. For seventeen years has been totally blind in both eyes, having a hard cataract. Was brought to the clinic, where Dr. Lewis operated, by displacing the lens. The operation was eminently successful. The patient did well, and yesterday I saw him,—five weeks after the operation,—and he could read distinctly, seeing as well from one eye as from the other.

CASE 2.—Mary O. S. ; age 41. Twelve years ago had a distressing labor of two days. "The forceps were applied several times. After two days the child's head was extracted, dead. It was "mashed in on both sides," and otherwise distorted. Examination *per vagina*, now shows uterus hypertrophied. The uterus and the bladder both lie in the mouth of the vagina. Uterus presses heavily on the bladder, which is below the symphysis, and between the rami. Incontinence of urine. Several minute vascular tumors of the urethra present. A colpeurynter was introduced, which gave her but little pain, but prevented her from making water. It was removed five hours after introduction, to enable her to urinate, but was immediately re-introduced ; was obliged to repeat the operation every two hours in order to pass her water. For one week the patient used the instrument faithfully, and the symptoms of incontinence of urine disappeared. Before its use, was obliged to pass her water nearly every five minutes.

The colpeurynter gives her but little pain. After wearing this instrument seven weeks, it was observed that it only did good while in use, and at the end of this time, whenever it was removed, the symptoms all reappeared with no diminiution in severity. Chloroform was accordingly administered, and the old cicatrices of the ruptured perineum were nicely trimmed and freshened with the scissors, and the edges then brought together with quilled sutures. A curious feature presented itself during the operation. At the time of the commencement of the inhalation of the chloroform, the colpeurynter was in the vagina. At the first clip of the scissors she experienced some pain, and evidently, in her semiconscious state, imagined she was in labor, and at once *commenced to bear down with all her force.* At about the usual intervals of labor pains, and coming on at regular intervals, she would bear down, pressing outward the colpeurynter, which she evidently supposed to be the *child's head.* As soon as this was *born*, she evidently seemed to experience very much relief, and in a few moments commenced to be excessively hysterical, laughing, sobbing and crying out at short intervals. Soon after the operation, the colpeurynter was again introduced, and the patient given ¼ gr. *Murph. Sulph.* The following day, although there had been some little infiltration of urine, still she was comfortable and doing well. Gave Dover's powder. On the ninth day all dressings were removed. The parts had united nicely, and the difficulty in urination had entirely subsided, and the patient was discharged *cured.*

E. R. Hutchins, M.D.

